# Joint Bacterial Traces in the Gut and Oral Cavity of Obesity Patients Provide Evidence for Saliva as a Rich Microbial Biomarker Source

**DOI:** 10.3390/nu17223527

**Published:** 2025-11-11

**Authors:** Jacqueline Rehner, Madline Gund, Sören L. Becker, Matthias Hannig, Stefan Rupf, Jörn M. Schattenberg, Andreas Keller, the IMAGINE Consortium, Leidy-Alejandra G. Molano, Verena Keller

**Affiliations:** 1 Institute of Medical Microbiology and Hygiene, Saarland University, 66421 Homburg, Germanysoeren.becker@uks.eu (S.L.B.); 2 Clinic of Operative Dentistry, Periodontology and Preventive Dentistry, Saarland University, 66421 Homburg, Germany; madline.gund@uks.eu (M.G.); matthias.hannig@uks.eu (M.H.); stefan.rupf@uks.eu (S.R.); 3 Department of Internal Medicine II, Saarland University, 66421 Homburg, Germany; joern.schattenberg@uks.eu; 4 PharmaScienceHub, 66123 Saarbrücken, Germany; andreas.keller@ccb.uni-saarland.de; 5 Clinical Bioinformatics, Saarland University, 66123 Saarbrücken, Germany; alejandra.gonzalez@ccb.uni-saarland.de; 6 Helmholtz Institute for Pharmaceutical Research Saarland, 66123 Saarbrücken, Germany

**Keywords:** microbiome, obesity, oral–gut axis, microbial biomarkers, metagenomics

## Abstract

**Background**: The human microbiome holds promise for identifying biomarkers and therapeutic targets. In obesity, interactions between oral and gut communities are increasingly implicated and end in organ injury. **Methods**: From the IMAGINE study, we analyzed 418 shotgun metagenomes from three specimen types (dental plaque (n = 143; 65 non-obese, 78 obese), saliva (n = 166; 75 non-obese, 91 obese), and stool (n = 109; 57 non-obese, 52 obese)) to compare site-specific microbial shifts between obese (BMI > 30 kg/m^2^) and non-obese individuals. Differential abundance was assessed with ANCOM-BC; effect sizes were summarized as Cohen’s d. **Results**: Across all samples, we detected 240 bacterial species in plaque, 229 in saliva, and 231 in stool, with 46 species present across all three sites. Absolute effect sizes were significantly larger in plaque (mean |d| = 0.26) and saliva (0.25) than in stool (0.21; *p* = 9 × 10^−3^). Several taxa showed an opposite directionality between oral and gut sites, including *Streptococcus salivarius* and *Bifidobacterium longum*, indicating site-specific associations. Notably, *Actinomyces* sp. and *Streptococcus* sp. exhibited promising effect sizes as diagnostic markers. **Conclusions**: The oral and gut microbiomes capture complementary obesity-related signals, with stronger shifts observed in oral sites. We suggest that integrating oral and gut profiling could enhance diagnostic and therapeutic strategies in obesity.

## 1. Introduction

Obesity is a multifactorial chronic disease resulting from complex interactions between genetic, metabolic, environmental, and behavioral factors [1]. Beyond excess body fat accumulation, it represents a systemic condition characterized by chronic low-grade inflammation, altered hormonal signaling, and metabolic dysfunction, ultimately predisposing individuals to type 2 diabetes, cardiovascular, renal and liver disease, and cancer [2,3]. Despite the central role of caloric imbalance, it has become increasingly evident that obesity cannot be fully explained by energy intake alone [4]. In recent years, the human microbiome has emerged as a key regulatory component of metabolic homeostasis, influencing nutrient absorption, lipid metabolism, bile acid turnover, and the immune system. The gut microbiota has been intensively studied in the context of obesity [4,5,6,7,8,9,10].

A comprehensive review emphasized that gut dysbiosis contributes to obesity pathogenesis through altered nutrient absorption, adiposity, and appetite regulation, suggesting microbiota-targeted interventions such as probiotics, prebiotics, synbiotics, and fecal microbiota transplantation as promising therapeutic strategies, although clinical evidence remains heterogeneous [5]. Intervention studies in adults with obesity further support the functional relevance of the gut microbiota. In the WLM3P trial, dietary modification combining caloric restriction, prebiotic supplementation, and time-restricted eating induced significant microbial shifts, particularly an increase in *Faecalibacterium* abundance, which correlated with reductions in fat mass and visceral adiposity, pointing to microbiota-mediated mechanisms of weight loss [7]. At the mechanistic level, metagenomic and multi-omics analyses have revealed direct links between microbiota composition, carbohydrate metabolism, and host insulin resistance. A large-scale study showed that microbial carbohydrate metabolism can contribute up to 10% of host energy extraction and is closely associated with insulin resistance and low-grade inflammation in obesity and prediabetes [8]. Complementary metabolomic approaches have expanded this understanding by identifying microbiota-derived metabolites that influence host metabolic pathways. An integrated mass-spectrometry pipeline demonstrated that gut bacteria produce diverse bioactive small molecules with direct effects on host physiology, including those implicated in metabolic disease and obesity [6]. Moreover, specific microbial metabolites can mechanistically drive obesity-related metabolic changes. In mouse models, dietary fructose was shown to be converted by gut microbes into acetate, which then fuels hepatic lipogenesis and contributes to fat accumulation, establishing a clear causal link between diet–microbiota interactions and obesity [10]. Finally, foundational work in human twins established that obesity is associated with reduced bacterial diversity and phylum-level alterations of the gut microbiota, notably an increased Firmicutes-to-Bacteroidetes ratio and functional deviations from the shared “core” microbiome, indicating that microbiome composition reflects and potentially contributes to host adiposity [9].

Most human microbiome studies in obesity focus exclusively on stool samples, as they are easily accessible and rich in microbial DNA. While fecal metagenomes provide valuable insight into intestinal microbial composition, they represent only a localized fraction of the human microbiota. The gastrointestinal tract forms a continuous axis beginning in the oral cavity, which harbors diverse microbial communities that interact with host metabolism through inflammatory and metabolic pathways. These oral microbiota, including saliva- and plaque-associated bacteria, may not only reflect systemic metabolic status but could actively contribute to disease mechanisms. Yet, their role in obesity remains comparatively underexplored. Thus, other easily accessible sample types, such as saliva and interdental plaque have gained attention for their potential to offer complementary diagnostic insights. Evidence suggests a key role of oral microbiota on metabolic and other diseases [11]. Moreover, a shift from the oral cavity to the gut with pro-inflammatory potential is well known [12]. The oral microbiome relates also to systemic conditions like Parkinson’s disease, where saliva-based analyses have shown promising diagnostic accuracy, often surpassing stool in early disease detection [13]. It is also known that the diseases periodontitis and diabetes are linked and have also an influence on the gut microbiome [14,15]. Similarly, rapidly developing sequencing technologies allow discovering pivotal information about host–microorganism interactions and health outcomes in general [16]. This underlines the utility of microbiota as a diagnostic reservoir and for therapeutic approach. These findings challenge the traditional stool-centric approach and advocate for a multi-sample strategy in microbiome diagnostics. Beyond the diagnostic repertoire, describing correlations between microbial community changes and disease states, the biosynthetic potential as natural producers [17] is considered towards understanding functional contexts of microbes on their hosts.

**Figure 1:**
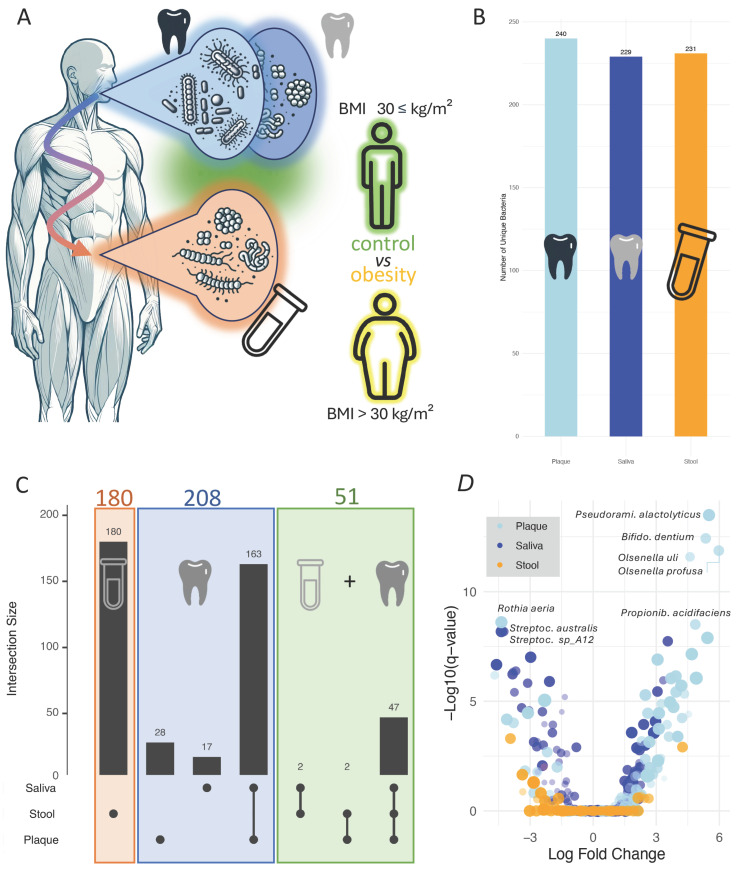
(**A**) Study set up. We set to compare gut and oral cavity microbiomes to find joint traces between the two body sites. (**B**) Number of species detected in the three sample types. (**C**) Upset-plot that provides an overview how the species discovered in the three sample types are distributed. (**D**) Volcano plot that presents the log fold changes versus the *p*-values for detecting obesity. Each dot corresponds to one bacterial species in one of the three sample types. The sample type is represented by the different colors.

Despite recent advancements, another significant challenge remains in microbiome research. One of the most pressing issues is the lack of standardized protocols for sample collection, storage, and processing. Variations in DNA extraction methods, for example, can lead to discrepancies in microbial diversity and composition, particularly affecting hard-to-lyse Gram-positive bacteria [18]. These methodological inconsistencies complicate cross-study comparisons and hinder the reproducibility of findings. Recognizing this we evaluated validated protocols to ensure data quality and comparability [19]. This methodological rigor is essential for translating microbiome research into clinical practice, where diagnostic tools must be both accurate and reproducible. Against this background, we initiated the IMAGINE [20] study (identification of microbial antibiotics to protect the physiologic microbiota at body surfaces) to address two key aims, i.e., (1) to systematically analyze the microbiomes of different body compartments in health and disease, and (2) to explore their potential as reservoirs of natural products, including biosynthetic gene clusters (BGCs), which may mediate microbe–host and microbe–microbe interactions. The IMAGINE study exemplifies one paradigm shift by employing a cross-disease, cross-sample analysis to explore microbial variations across different body sites and health conditions [20]. This approach revealed notable site-specific differences in microbial composition and functionality, reinforcing the diagnostic value of integrating multiple sample types. Such findings underscore the need to move beyond single-sample studies to capture the complex interplay between microbial communities at different anatomical sites. While this study identified site-specific trajectories for many diseases across stool, saliva, and interdental plaque, offering a more nuanced understanding of disease-associated dysbiosis, obesity was not considered in detail. In analyzing the highly standardized data from the IMAGINE study we identified distinct patterns in the microbial composition within the oral samples (interdental plaque and saliva) and stool samples of patients diagnosed with obesity. The objective of this study was to assess whether the oral (saliva/plaque) or gut microbiota provide greater complementary diagnostic value in obesity. While microbial profiles are not intended to replace BMI, they may serve as additional measure to assess metabolic health. A secondary aim was to determine whether dysregulation of specific species occurs in both compartments, potentially supporting improved treatment monitoring in obesity (Figure 1A). With our study, we test the hypothesis that oral microbial communities, particularly from plaque and saliva, might exhibit stronger or more specific obesity-related signatures than the gut microbiota, given their close interaction with dietary exposure and systemic inflammatory pathways.

## 2. Materials and Methods

Clinical sampling: Detailed procedures are described in the IMAGINE main manuscript [20]. In brief, clinical samples were collected from participants at Saarland University Medical Center in Homburg, Germany, following written informed consent. Ethical approval for the study was granted by the ethics committee of the local medical association (Ärztekammer des Saarlandes, ID: 131/20). Inclusion criteria were as follows: all patients with the relevant diagnosis obesity (BMI > 30 kg/m^2^) were eligible for participation. Exclusion criteria were: Missing informed consent, acute exacerbation of the underlying condition, acute unrelated illness, age under 18 years, or ongoing pregnancy. Each participant underwent a thorough medical and dental examination to identify diseases of interest, and an in-depth medical history was recorded, including relevant factors such as medication, diet, activity levels, smoking, alcohol consumption, and co-morbidities. Samples included saliva, interdental plaque, conjunctiva swabs, throat swabs, stool, and skin swabs from the forehead and arm, as well as affected areas for participants with Acne inversa. The diagnostic procedures and sample collection were performed as described in the original study. Overall, we included 418 shotgun metagenomes from the three specimen types (dental plaque (n = 143; 65 non-obese, 78 obese), saliva (n = 166; 75 non-obese, 91 obese), and stool (n = 109; 57 non-obese, 52 obese)).

DNA extraction, library prep and sequencing: Detailed DNA extraction and sequencing protocols are described in the IMAGINE main manuscript [20]. In brief, DNA extraction was performed using the Qiagen QiAamp Microbiome Kit (Qiagen, Hilden, Germany) following the manufacturer’s protocol. Extracted DNA from all samples was sent to Novogene Company Limited (Cambridge, UK) for metagenomic library preparation and paired-end (PE150) sequencing using the Illumina HiSeq platform. To ensure high-quality data, we carried out a very stringent quality filtering across the whole pipeline and excluded 1552 samples within the original IMAGINE analysis [20]. For this study we used the cleaned data.

Primary data analysis: Detailed primary data analysis procedures are described in the IMAGINE main manuscript [20]. Sequencing data underwent initial quality control and host decontamination using KneadData (v0.7.4) [21] and applied an additional filtering step using sra-human-scrubber [22]. Paired-end reads were only kept if none of the read pairs mapped to the human reference. MetaPhlAn3 [21] was applied to profile quality-controlled samples and relative counts were rescaled to absolute counts. To estimate alpha diversity, Shannon diversity was used. Differential abundance analyses were performed using ANCOM-BC (Analysis of Composition of Microbiomes with Bias Correction) [23]. Analyses were conducted separately for each disease group included in IMAGINE and each specimen type (stool, saliva, plaque). For each comparison, *p*-values were adjusted to control the false discovery rate (FDR), considering the number of species tested per specimen type. A species was considered significantly associated with disease if the FDR-adjusted q-value was below 0.05.

Downstream data analysis: Downstream bioinformatics analysis was conducted to extract and visualize meaningful insights from the microbial metagenomic data that we extracted from IMAGINE. The dataset consisted of taxonomic, statistical, and metadata information for various specimens, including stool, saliva, and interdental plaque. Data preparation, visualization, and statistical evaluation were carried out using R (Version 4.5.1), with detailed steps outlined below. Initially, IMAGINE data were imported and preprocessed. The dataset was read as a matrix, with separate annotation and numerical matrices created for metadata (e.g., specimen types, taxonomic species) and statistical values (e.g., *p*-values, fold changes, effect sizes). Filtering focused on samples flagged as “all” in the comparison column, excluding other entries. Volcano plots were generated to visualize the relationship between log-fold changes and *p*-values for specific group comparisons, such as Obesity versus Healthy. For each microbial species, the effect size of abundance differences between obese and non-obese individuals was quantified using Cohen’s D. This statistic measures the standardized mean difference between two groups and was preferred over *p*-values for its robustness to differences in cohort size. Cohen’s D was calculated as the difference between the mean relative abundance in the obese group and the mean relative abundance in the non-obese group, divided by the pooled standard deviation of both groups. The pooled standard deviation was derived from the within-group variances, weighted by their respective sample sizes. Scatterplots compared effect sizes across specimen types at the species level with regression lines quantifying the relationships between effect sizes in saliva versus plaque, saliva versus stool, and plaque versus stool. For species present across all three specimen types, dot plots were created. These displayed effect sizes for each specimen type, with color coding and point sizes indicating effect size magnitude. Species were ordered by their average effect size across specimen types, highlighting dominant taxa. Back-to- back histograms are generated to illustrate the distribution of effect sizes for plaque, saliva, and stool. Similarly, heatmaps visualized −log10-transformed q-values for significant bacterial species, categorized by specimen type. For computing beeswarm plots with significance values the ggbetweenstats package has been used [24]. Lastly, UpSet plots depicted the overlap of species presence across stool, saliva, and plaque, providing an overview of shared and unique taxonomic features.

Literature Search: To assess the plausibility of our findings, we conducted a comprehensive PubMed search. Each of 35 species identified across all three sample types was queried using a combination of terms: “plaque”, “saliva”, and “stool” (representing the three specimen types), paired with “obesity”. In addition, the disease terms “Crohn’s disease”, “Colitis”, “Caries”, and “Periodontitis” were searched to obtain the background distribution. This approach yielded a total of 35 × 3 × 5 = 525 individual queries, systematically designed to evaluate the associations between species, specimen types, and disease contexts through an evidence-based review of the existing literature.

## 3. Results

### 3.1. Microbiome-Disease Associations Vary Across Specimen Types with Inter-Site Overlap

The first step of our analysis was to establish a baseline set of bacterial species across the three specimen types. To this end, we extracted all species from the IMAGINE cohort that were tested for disease associations using ANCOM-BC, independent of their *p*-values or effect sizes. This inclusive approach yielded a set of 700 species that showed an association with at least one disease in at least one specimen type (stool, saliva, or plaque). Notably, at this stage we did not apply any significance threshold; rather, the goal was to capture the full landscape of tested associations to serve as a reference for subsequent filtering and comparative analyses. These associations were distributed evenly among the specimen types: interdental plaque samples showed the highest number with 240 associations, followed by stool with 231, and saliva with 229 (Figure 1B). It is important to highlight that the 700 associations do not correspond to 700 distinct species, as some species were associated with diseases in more than one specimen type. In total, the 700 associations represented 439 unique species. Of these, 225 species were specific to a single specimen type, 167 were shared between any two specimen types, and 47 species (10.7%) were identified in all three habitats. When stratified by anatomical region, 208 species were detected exclusively in the oral cavity, while 180 were specific to stool. An additional 57 species were shared between the oral cavity and stool (Figure 1C). This overlap suggests that for a subset of bacterial species an interplay between different microbiome niches across the body exist that might have an impact on their role in disease associations. In this context, the oral–gut axis is among the best understood ones [25], although functional cascades are not fully explored yet. Given the focus on obesity, we restricted our analysis on 629 associations between the three specimen types and obesity. A volcano plot comparing log fold change and q-values (Figure 1D) revealed a predominantly oral signature with the four most significant associations all in plaque: *Pseudoramibacter alactolyticus* (q = 3.27 × 10^−14^; d = 0.60), *Bifidobacterium dentium* (q = 3.77 × 10^−13^; d = 0.39), *Olsenella uli* (q = 1.37 × 10^−12^; d = 0.41), and *Olsenella profusa* (q = 2.63 × 10^−12^; d = 0.29). Beyond these, several periodontal/pathobiont-enriched taxa in plaque showed moderate-to-large positive effect sizes, including *Tannerella forsythia* (q = 8.82 × 10^−7^; d = 0.73), *Desulfobulbus oralis* (q = 1.28 × 10^−8^; d = 0.64), *Eubacterium nodatum* (q = 1.13 × 10^−5^; d = 0.63), *Treponema vincentii* (q = 5.16 × 10^−6^; d = 0.54), *Dialister pneumosintes* (q = 1.84 × 10^−5^; d = 0.58), and *Bulleidia extructa* (q = 1.25 × 10^−7^; d = 0.60). In contrast, oral commensals were depleted, notably *Rothia aeria* in plaque (q = 2.47 × 10^−9^; d = −0.59) and saliva (q = 4.14 × 10^−7^; d = −0.25), alongside multiple salivary *Streptococcus* spp. with negative effects (e.g., *S. australis* q = 6.59 × 10^−9^; d = −0.48, *Streptococcus HMSC067H01* q = 9.78 × 10^−8^; d = −0.48, *S. infantis* q = 1.25 × 10^−6^; d = −0.44). Importantly, we observed cross-site consistency for several taxa: *Bifidobacterium dentium* was enriched in both plaque (q = 3.77 × 10^−13^; d = 0.39) and saliva (q = 1.11 × 10^−6^; d = 0.18); *Propionibacterium acidifaciens* was positive in plaque (q = 3.13 × 10^−9^; d = 0.40) and saliva (q = 1.83 × 10^−8^; d = 0.37); *Tannerella forsythia* was increased in both plaque (q = 8.82 × 10^−7^; d = 0.73) and saliva (q = 8.38 × 10^−5^; d = 0.50); and *Fretibacterium fastidiosum* showed concordant enrichment in plaque (q = 1.93 × 10^−6^; d = 0.60) and saliva (q = 3.59 × 10^−6^; d = 0.39). Conversely, *Actinomyces massiliensis* decreased in both sites (plaque q = 9.02 × 10^−6^; d = −0.76; saliva q = 1.49 × 10^−6^; d = −0.27), as did *Rothia aeria* (see above). Additional salivary depletions included *Neisseria flavescens* (q = 4.43 × 10^−7^; d = −0.14), *Oribacterium parvum* (q = 5.86 × 10^−7^; d = −0.37), and *Eubacterium sulci* (q = 1.99 × 10^−5^; d= −0.28). Notably, stool did not yield taxa at comparable significance levels within this set, reinforcing the dominance of oral-site signals in our volcano-derived associations. While these findings underscore the diverse and dynamic microbial changes occurring in stool, plaque, and saliva, they highlight the importance of including multiple specimen types to obtain a comprehensive picture of microbiome–disease interactions. We thus initiated a direct comparison between the gut and oral cavity.

### 3.2. Oral Samples Display Significantly Higher Effect Sizes as Compared to Stool Samples

**Figure 2:**
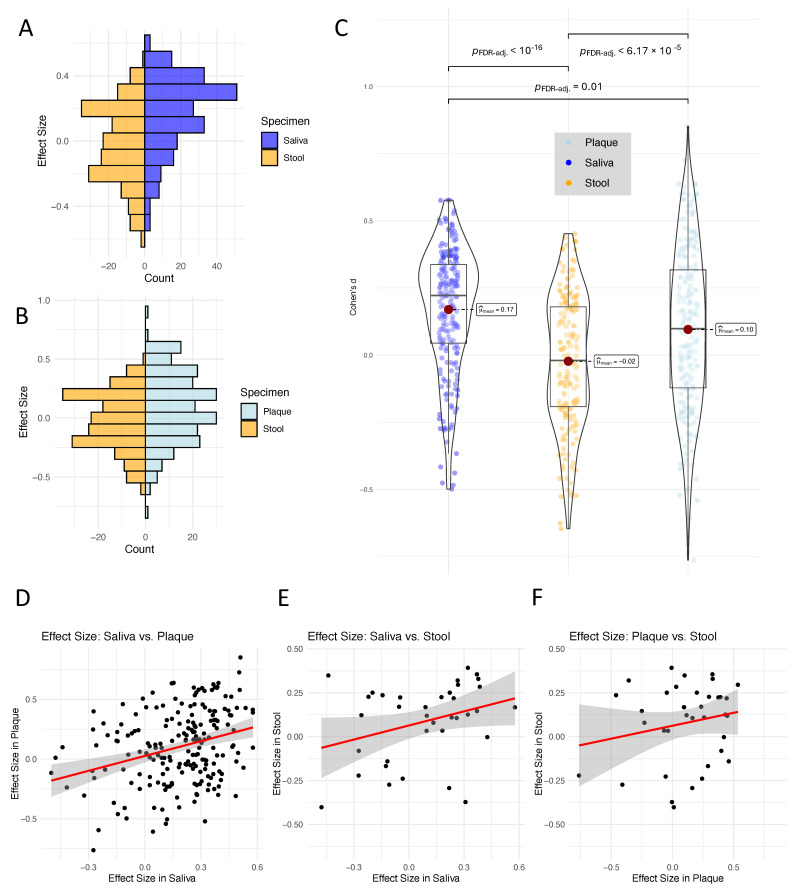
(**A**) Back-to-back histogram comparing the effect sizes between saliva (blue) and stool (orange) species. (**B**) Back-to-back histogram comparing the effect sizes between plaque (blue) and stool (orange) species. (**C**) Direct comparison of the effect sizes as beeswarm plots. Mean values and significance values are used to annotate the beeswarm plot and box plots per distribution are shown in the background for each sample type. (**D**) Scatter plot comparing the effect sizes in saliva to those in plaque. The regression is presented as red lines. The gray area represents the respective confidence interval. (**E**) Same panel as in (**D**) but for saliva and stool. (**F**) Same panel as in (**D**) for plaque and stool.

Because *p*-values can be misleading or show only an incomplete picture of the actual differences [26,27,28] (e.g., they do not convey the magnitude of an effect, can be influenced by sample size, and are susceptible to misinterpretation) and fold changes can likewise be misleading (e.g., due to their sensitivity to variability in data and potential to overemphasize minor differences) we have chosen to focus on effect sizes, which provide a more balanced and interpretable measure of the strength of associations, offering a clearer understanding of the practical significance of our findings. First, we computed back-to-back histograms to compare the effect sizes for diagnosing obesity samples in stool versus saliva (Figure 2A) and stool versus interdental plaque (Figure 2B). These histograms revealed a shift towards higher effect sizes in saliva and plaque compared to stool. To further explore these differences, we utilized beeswarm plots to visualize the effect sizes across the three specimen types and carried out statistical comparisons using the Games–Howell post hoc test. Our approach is appropriate for scenarios with unequal group variances or differing sample sizes, ensuring robust pairwise comparisons. The analysis revealed that plaque exhibited significantly higher effect sizes (mean effect size of 0.17) compared to saliva (mean of 0.1) and stool (mean of −0.02) (Figure 2C). Shifting to the absolute values of the effect size, the oral cavity does not display a significant difference between each other but a significantly higher effect sizes as compared to stool. These findings underscore the distinctiveness of saliva and plaque in capturing biological effects relevant to obesity and emphasize its potential as a diagnostic specimen.

In the previous analyses, we did not explicitly address direct relationships of individual bacterial species across specimen types. To explore these relationships in greater detail, we computed scatter plots to compare the effect sizes of each species between pairs of specimen types. Specifically, we compared species effect sizes between saliva and interdental plaque (Figure 2D), saliva and stool (Figure 2E), and interdental plaque and stool (Figure 2F), allowing for a direct species-by-species assessment of the effect sizes, highlighting potential correlations and trends between them. The results demonstrate a significant positive correlation between saliva and interdental plaque, indicating a shared microbial signature in these oral cavity specimens. Specifically, the pairwise comparisons of effect sizes across body sites revealed a moderate and highly significant correlation between saliva and plaque (Pearson r = 0.30, *p* = 1.7 × 10^−5^; Spearman r = 0.28, *p* = 8.2 × 10^−5^). The correlation between saliva and stool was similar but did not reach significance, partially driven by a lower number of shared species (Pearson r = 0.32, *p* = 0.057; Spearman r = 0.29, *p* = 0.090). For plaque and stool, the association was lower and nonsignificant (Pearson r = 0.20, *p* = 0.25; Spearman r = 0.13, *p* = 0.44). This suggests that species showing higher effect sizes in saliva are likely to exhibit similar trends in interdental plaque, reflecting their common origin within the oral microbiome. In contrast, when comparing saliva to stool, we observed less apparent correlation. Similarly, for interdental plaque versus stool, there is at most a slight positive correlation, which may reflect the influence of shared bacterial taxa between these two sites but with significant variability. While this distribution pattern might be expected and aligns with established assumptions about microbial compartmentalization, we here provide a systematic and quantitative confirmation across matched multi-site samples, thereby formalizing this assumption. These results also motivate a more detailed consideration of the shared species presented in the next section, as they highlight that overlap in species presence does not necessarily translate into consistent disease associations: several species are shared across specimen types, yet their effect sizes differ substantially, sometimes potentially pointing even in opposite directions between the gut and the oral cavity. This divergence reinforces the importance of considering specimen-specific factors when analyzing microbial data and selecting specimen types for diagnostic purposes. We thus aimed to directly compare bacterial species that occur across all three specimen types. This approach provides a focused analysis of species with broad representation across different microbiomes, allowing for a more integrated perspective. In total, we identified 36 of such cases. Among these, one entry corresponds to unknown species, included for the sake of completeness. Excluding this single exception, the analysis concentrated on 35 well-characterized bacterial species. This targeted approach provides valuable insights into the dynamics of these species and their potential relevance for understanding microbial patterns in relation to disease.

### 3.3. Cross-Site Consistency and Divergence of Obesity-Associated Microbial Signatures

**Figure 3:**
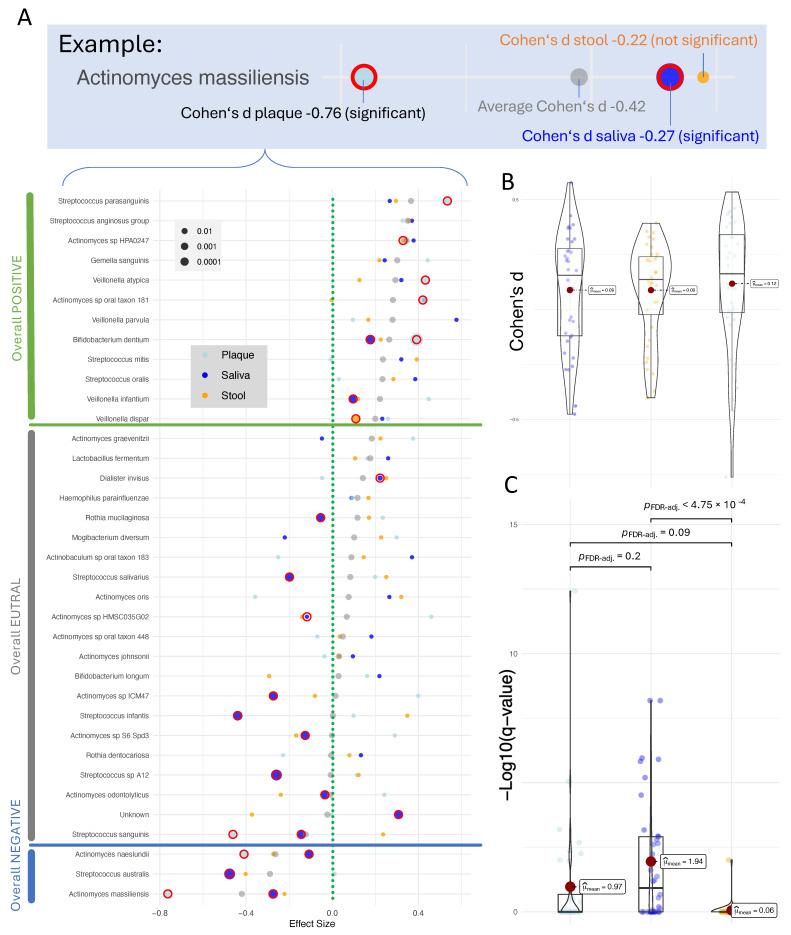
(**A**) Effect sizes and q-values of bacterial species across saliva, plaque, and stool specimens. Each dot represents the effect size of a bacterial species in one of the three specimen types (saliva: blue, plaque: light blue, stool: orange). Gray dots indicate the average effect size of each species across all three specimen types. The species are ordered by their average effect size in ascending order. The size of the colored dots reflects the q-value from ANCOM-BC. q-values below 0.05 are highlighted by red circles. The top of the panel presents an example of one species. The green dashed vertical line marks an effect size of 0, the blue and green horizonal lines distinguish average low effect size (blue) and average high effect size (green) species. This visualization highlights the variability and trends in bacterial species’ contributions across the different specimen types. (**B**) Direct comparison of the effect sizes as beeswarm plots. Mean values and are used to annotate the beeswarm plot and box plots per distribution are shown in the background for each sample type. (**C**) Direct comparison of the q-values as beeswarm plots. Mean values and significance values are used to annotate the beeswarm plot and box plots per distribution.

To identify consistent microbial trends across different body sites, we compared effect sizes (Cohen’s d) of the top taxa sorted by their mean absolute effect across saliva, plaque, and stool (Figure 3A). Four of the five top species showed concordant directionality across all specimen types, suggesting systemic or multisite associations with obesity, whereas the fifth species demonstrated site-specific divergence. The strongest and most consistent reduction was observed for *Actinomyces massiliensis* (Saliva: d = −0.27; Plaque: −0.76; Stool: −0.22), which showed negative effect sizes in all sites, indicating a generalized depletion in obese individuals. This broad pattern supports the hypothesis that *Actinomyces* species, typically commensal oral bacteria with roles in biofilm stability and mucosal health, may be suppressed under obesity-associated inflammatory or metabolic conditions. The next three species, *Streptococcus parasanguinis* (d = 0.27/0.53/0.30), *Streptococcus anginosus* group (d = 0.37/0.33/0.36), and *Actinomyces* sp. *HPA0247* (d = 0.38/0.33/0.33), each displayed moderate, positive, and highly consistent effect sizes, indicating a uniform enrichment across oral and gut environments in obese participants. These taxa belong to facultative anaerobic oral streptococci and actinomyces, which are often associated with mucosal colonization and early biofilm formation. Their parallel behavior across sites may reflect either oral–gut microbial transfer or shared ecological drivers such as dietary carbohydrate availability or altered immune regulation.

In contrast, *Actinomyces oris* exhibited a divergent pattern (Saliva: d = 0.27; Plaque: −0.36; Stool: 0.32), showing enrichment in saliva and stool but a decrease in plaque. This heterogeneity suggests that even closely related *Actinomyces* species may occupy distinct ecological niches within the oral cavity and gut, responding differently to obesity-related host conditions such as pH shifts, nutrient gradients, or salivary composition. A similar divergence was observed among *Streptococcus* taxa, particularly *Streptococcus australis* (Saliva: d = −0.48; Plaque: 0.01; Stool: −0.40) and *Streptococcus infantis* (Saliva: d = −0.44; Plaque: 0.10; Stool: 0.35). Both species showed strong negative associations in the oral cavity, whereas *S. infantis* was markedly increased in stool, suggesting opposite ecological adaptations in oral versus intestinal environments. While oral streptococci are key contributors to carbohydrate metabolism and biofilm integrity in the mouth, their presence in stool may reflect either transient passage or successful colonization under the nutrient-rich conditions associated with obesity. The contrasting directionality for *S. infantis* further supports the notion that some streptococcal species may play distinct, site-specific roles, potentially beneficial or opportunistic, depending on their metabolic context and interaction with the local immune environment. Two other remarkable species displayed distinct distribution patterns across specimen types. *Streptococcus sanguinis* (Saliva: d = −0.14; Plaque: −0.46; Stool: 0.24) showed a consistent decrease in both oral habitats but an increase in stool, representing an opposite trend between the oral cavity and the gut. Another distinct pattern was observed for *Bifidobacterium longum* (Saliva: d = 0.22; Plaque: 0.16; Stool: −0.29), which was increased in the oral cavity but decreased in stool, indicating an inverse oral–gut relationship. In a similar manner, *Actinomyces* sp. oral taxon 181 (Saliva: d = 0.43; Plaque: 0.42; Stool: 0) was elevated in both oral specimen types, while remaining nearly unchanged in stool. These patterns further illustrate the presence of species with either concordant or divergent abundance shifts across different body sites. In summary, the cross-compartment comparison revealed multiple distinct patterns of microbial abundance shifts between obese and non-obese individuals. Some species were increased across all specimen types, such as *Streptococcus parasanguinis*, while others were consistently decreased, including *Actinomyces massiliensis*. Certain taxa showed increases restricted to the oral cavity, for instance *Actinomyces* sp. oral taxon 181, whereas others were decreased only in the oral samples, such as *Streptococcus australis.* Additional examples displayed concordant changes between stool and only one of the oral habitats, for example, *Actinomyces oris*, which was increased in saliva and stool but reduced in plaque. These diverse distribution patterns highlight both shared and site-specific microbial responses to obesity across the oral–gut axis. To further evaluate whether these trends correspond to general differences in effect magnitude between specimen types, we next compared the overall distributions of effect sizes across saliva, plaque, and stool to assess whether the pattern observed in Figure 2C is consistently reflected in the current subset of bacteria.

In contrast to the analysis including all detected species, where considerably higher effect sizes were observed in the oral samples, the distribution of effect sizes among the shared species appears more balanced across specimen types. As shown in the violin plots, the median absolute effect sizes are similar for plaque (d = 0.09), saliva (d = 0.09), and stool (d = 0.12), indicating that obesity-related microbial shifts in these overlapping taxa are of comparable magnitude across body sites (Figure 3B). This suggests that, while oral samples generally display stronger compositional differences when all taxa are considered, the subset of species present in all three habitats contributes more evenly to obesity-associated variation. For completeness, we also compared the distribution of −log10-transformed q-values across specimen types (Figure 3C). The mean values were considerably higher in the oral samples than in stool, with plaque showing a mean of 1.94, saliva 0.97, and stool only 0.06. This indicates that microbial associations with obesity reached higher levels of statistical significance in the oral habitats. However, these differences should be interpreted considering the underlying sample sizes, as the oral datasets contained substantially more samples than stool, which likely contributed to the stronger statistical signals observed. Together with the more balanced effect sizes among shared taxa, these results suggest that both effect magnitude and cohort size influence detectability. Overall, they highlight that integrating data from multiple body sites offers a more comprehensive understanding of microbiome alterations in obesity, allowing systemic and site-specific components to be distinguished. However, for such markers, it is important to understand their potential functionality and whether they are already described and associate to obesity or other oral and intestinal disease. While a deep functional characterization is beyond the scope of the present work, we made use of the existing and extensive literature on metagenomics in relation to human diseases.

### 3.4. Literature Mining Supports Relevance of Identified Species Across Diseases and Sites

**Figure 4:**
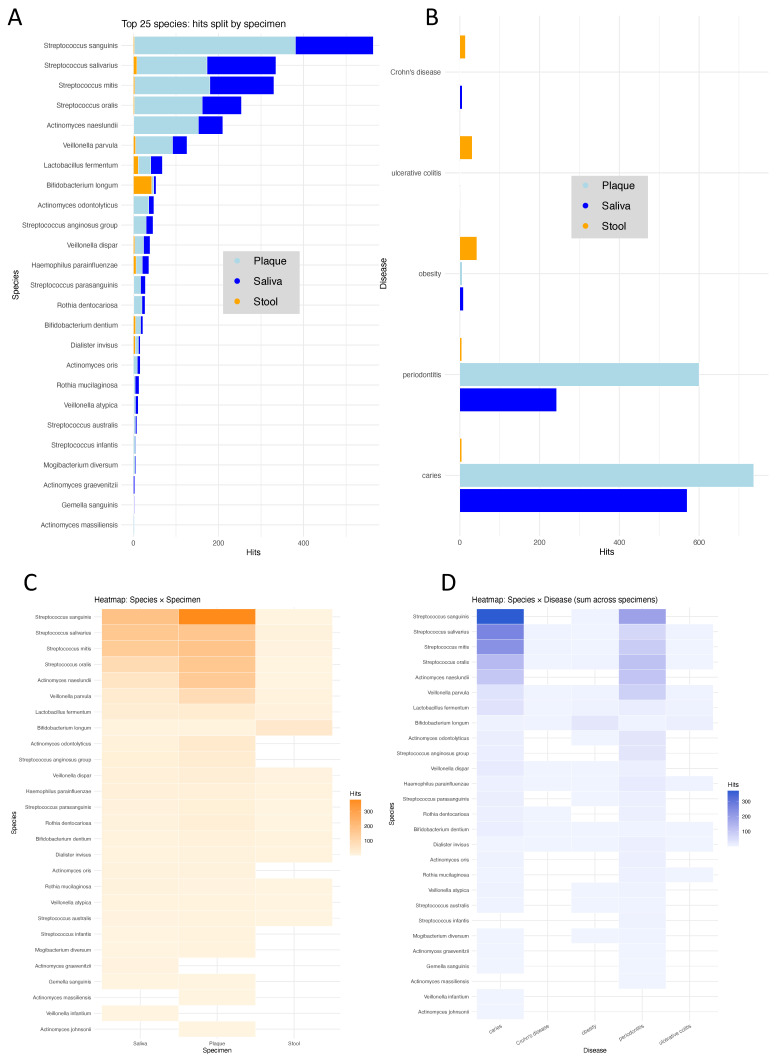
(**A**) Top 25 microbial species identified in the literature search, grouped by specimen type (saliva, plaque, stool). Most frequently reported species belong to the genera Streptococcus and Actinomyces, with predominant representation in oral samples. (**B**) Number of literature hits grouped by disease category. Caries and periodontitis dominate the published microbiome studies, while obesity, Crohn’s disease, and ulcerative colitis are represented by fewer but distinct associations. (**C**) Heatmap showing the distribution of literature hits per species across specimen types. Oral taxa such as Streptococcus sanguinis and Streptococcus salivarius show the strongest enrichment in plaque and saliva, whereas only few species, including Bifidobacterium longum, are also found in stool studies. (**D**) Heatmap summarizing literature associations by species and disease. Oral species are primarily linked to caries and periodontitis, while Bifidobacterium longum and Rothia mucilaginosa also appear in obesity and inflammatory bowel disease, indicating cross-compartment relevance.

The systematic literature search described in the Methods Section provided a comprehensive framework to evaluate potential associations between microbial species, specimen types, and disease contexts. Of the 525 PubMed queries performed, 147 returned at least one relevant hit. First, we grouped these results by species and specimen type, illustrating which taxa have been most frequently studied in relation to obesity and microbiome research (Figure 4A). The eight most frequently represented species were *Streptococcus sanguinis*, *Streptococcus salivarius*, *Streptococcus mitis*, *Streptococcus oris*, *Actinomyces naeslundii*, *Veillonella parvula*, *Lactobacillus fermentum*, and *Bifidobacterium longum*. These species were predominantly reported in the context of the oral cavity, consistent with their known ecological niches in saliva and dental plaque. However, for all of them, at least some publications also mentioned findings from stool or gut microbiome studies, indicating that these taxa are not exclusively confined to oral habitats. Notably, *Bifidobacterium longum* appeared across multiple specimen types and disease contexts, reflecting its well-established relevance for metabolic health and obesity-related research. We next grouped the literature hits by disease context (Figure 4B), including obesity, caries, and periodontitis as frequent conditions investigated in oral microbiome research, and Crohn’s disease and ulcerative colitis as common diseases in gut microbiomics. The resulting distribution shows that oral diseases dominate the literature, accounting for most reported associations. Both conditions yielded several hundred MEDLINE/PubMed hits, far exceeding the numbers retrieved for obesity or intestinal diseases. In contrast, obesity appeared in fewer studies, and inflammatory bowel diseases such as Crohn’s disease and ulcerative colitis were represented by only a small number of entries. This pattern underscores the strong research focus on oral microbiome alterations in dental pathologies compared to systemic metabolic or intestinal diseases, highlighting that obesity-related microbiome research in oral specimens remains comparatively underexplored. The enrichment of hits for obesity compared to the intestinal diseases, however, highlights the relevance of our findings for obesity, the focus of this study, and supports that several of the identified taxa have previously been linked to metabolic alterations rather than intestinal inflammation. Finally, we directly compared the literature evidence across species and contexts using two heatmaps (Figure 4C, species × specimen type, and Figure 4D, species × disease). Literature coverage by specimen type confirms that most taxa with the highest number of hits, such as *Streptococcus sanguinis*, *Streptococcus salivarius*, *Streptococcus mitis*, and *Actinomyces naeslundii*, are predominantly associated with oral samples, particularly plaque. Only a few species, including *Bifidobacterium longum* and *Lactobacillus fermentum*, also appear with relevant frequencies in stool studies. This pattern confirms that most existing research has focused on oral microbiome habitats, while cross-compartment studies remain limited. Summarizing literature associations by disease context, *Streptococcus* and *Actinomyces* species again dominate in oral diseases such as caries and periodontitis, whereas *Bifidobacterium longum* and *Rothia mucilaginosa* are among the taxa frequently mentioned in metabolic or intestinal conditions, including obesity and inflammatory bowel disease. Of note, many of the species are described however with different species and different specimen types, demonstrating that those are rather unspecific for any of the diseases. The overlap across these conditions further supports the role of several oral commensals as potential links between local dysbiosis and systemic metabolic disturbances.

## 4. Discussion

Human microbiota are increasingly recognized as a key player in maintaining metabolic health and contributing to disease initiation and progression [29]. Dysbiosis, or microbial imbalance, has been implicated in various conditions. While the gut microbiota has been extensively studied in this context, other body sites, such as the oral cavity, are gaining attention for their potential roles in systemic and localized inflammation [30]. This study aimed to comprehensively evaluate bacterial contributions to obesity across multiple specimen types, focusing on saliva, interdental plaque, and stool, to capture the diversity and specificity of microbial shifts. In the light of the high relevance of this topic, multiple studies related to the gut and saliva microbiome in diseases, in general, were summarized in a recent review on the gut–oral axis in health and diseases [25]. Often, the focus is either on the one or the other specimen types, using other sequencing strategies such as 16 s sequencing and focus on specific conditions such as lifestyle factors or therapies. While our study we tried to add to the current knowledge by having gut and saliva/plaque samples from the same patient collective using deep metagenomic sequencing.

Our results support the hypothesis that the patterns of interaction between oral and gut microbiota extend beyond obesity and indicate a broader role in systemic health and disease, as highlighted by multiple recent studies. Notably, the convergence of evidence from diverse conditions, including liver function recovery, cardiovascular diseases, and hepatic encephalopathy, emphasizes the intricate relationship between the microbiota of different body sites and systemic health [31,32]. *Bifidobacterium longum*, a key gut microbe, has been implicated in postoperative liver function recovery for hepatocellular carcinoma (HCC). A clinical trial demonstrated that oral administration of a probiotic cocktail containing *B. longum* reduced delayed recovery rates, shortened hospital stays, and improved overall survival. These benefits were linked to diminished liver inflammation, reduced fibrosis, and hepatocyte proliferation, underscoring the therapeutic potential of gut microbiota modulation [33]. In our study, this species is enriched in the oral cavity while we find it depleted in the stool samples of patients.

The connection between oral and gut microbiota is further demonstrated in cardiovascular diseases such as acute myocardial infarction (AMI). Oral species, including *Streptococcus oralis*, *Streptococcus parasanguinis*, and *Streptococcus salivarius*, have been shown to colonize the gut and aggravate AMI in murine models. These findings suggest that monitoring and controlling specific oral bacteria could represent a novel therapeutic avenue for cardiovascular diseases [34]. In our study, we find *Streptococcus oralis* highly significantly depleted in saliva, while it is not changed in plaque and moderately depleted in stool samples. *Streptococcus salivarius* is significantly depleted in saliva but enriched in plaque and stool samples. In contrast, *Streptococcus parasanguinis* is the overall most enriched species across specimen types with effect sizes of d = 0.27 in saliva, d = 0.53 in plaque and d = 0.30 in stool samples. These complex patterns indicate the importance of comparing the species in different specimen types of the same probands.

In sum, several microbial species identified with high effect sizes, enriched or depleted, in our analysis may play active roles in disease processes rather than merely reflecting dysbiosis but at the same time calling for experimental validation. To elucidate potential mechanisms, future research should include functional studies such as in vitro co-culture systems with epithelial or immune cells to assess microbial impact on barrier integrity, cytokine production, or immune activation. Additionally, gnotobiotic or antibiotic-treated animal models could be used to test causal relationships between specific taxa and colitis-like inflammation. Metatranscriptomic analyses may further help uncover niche-specific gene expression patterns, such as pro-inflammatory metabolite production or epithelial adhesion capabilities, which could explain differential associations across body sites. Such approaches would provide mechanistic insights that complement our observational findings.

In interpreting our data, it is essential to consider the complexity arising from the dynamic nature of the microbiome, which can be influenced by factors such as diet [35], medication [36], and disease state. A growing body of evidence suggests that not only diet composition but also the timing of nutrient intake can modulate host metabolism via the gut microbiome, emphasizing the dynamic interplay between dietary patterns, microbial functions, and metabolic health [37]. Additionally, different fiber types selectively shape microbial communities and metabolic functions along the gastrointestinal tract in goats [38]. As a last example, exclusive enteral nutrition (EEN), a first-line therapy for pediatric Crohn’s disease, has been shown to induce significant changes in gut microbiota, including shifts in strain-level dynamics and metabolite profiles [39]. Indirectly related to nutrition, studies highlight how gut microbiome composition and metabolic potential differ with host body size, suggesting that microbial adaptations may influence nutrient extraction efficiency and dietary requirements across species [37]. Respective studies highlight the importance of considering dietary context and microbial niche when interpreting metagenomic signatures. With respect to therapies, recent studies have shown that 5-aminosalicylic acid (5-ASA) not only reduces intestinal inflammation but also modulates the gut microbiota and supports barrier function, highlighting its dual role in immune regulation and microbial homeostasis [40,41]. Such findings emphasize the microbiome’s responsiveness to therapeutic interventions and its potential role in personalized medicine. However, they also underscore the need for longitudinal studies to account for temporal variations in microbial composition and function. In addition to its diagnostic potential, the microbiome offers valuable insights into disease mechanisms.

It is important to acknowledge limitations and suggest future research directions. One limitation is the cohort, which may not fully represent the variability of microbiota compositions across diverse populations or account for significant interindividual differences. Larger and more diverse datasets, encompassing tens of thousands of samples, are crucial to validate our findings and explore broader implications. Moreover, our analysis was complicated by uneven group sizes across different specimen types and patient groups. To address this limitation and ensure robust biological interpretation, we prioritized effect sizes over strict statistical significance thresholds (e.g., *p*-values). Additionally, while our cross-disease approach allowed us to identify common microbial patterns, it may have oversimplified distinct mechanisms underlying specific diseases. The role of comorbidities, which can significantly influence microbiota profiles and disease trajectories, is another grant challenge in respective studies. Especially a link between obesity and oral diseases is well established [42,43,44], making oral diseases a confounder for obesity profiles. Similarly, neither the plaque nor saliva microbiome are independent from each other and the gut microbiome is statistically not independent from the oral microbiota. Especially for diagnostic tests, those relationships might be considered. Moreover, future studies with a longitudinal design are essential to uncover how microbiota changes dynamically over time and how these changes correlate with disease progression or remission. A key limitation of this study is the absence of adjustment for potential confounding factors that are known to influence the microbiome in obesity. Variables such as dietary composition, medication use (notably antibiotics and metformin), metabolic comorbidities including type 2 diabetes, and oral health conditions such as periodontitis or caries may have contributed to the observed microbial patterns. While the IMAGINE dataset provides standardized sampling procedures and extensive metadata, it was not designed to capture detailed dietary intake or medication histories in detail. Consequently, residual confounding cannot be excluded, and the associations reported here should be interpreted as obesity-related rather than strictly obesity-specific. Future studies should integrate targeted nutritional and clinical data and employ longitudinal designs to disentangle causal effects from co-occurring factors. In this context, broad population-based resources such as IMAGINE could be complemented by more focused mechanistic studies addressing specific dietary or therapeutic interventions, as demonstrated in recent metagenomic and nutrition-focused investigations from our group and others. To address limitations, we put our results in the context of the literature. But the reliance on PubMed searches for validation introduces its own set of challenges. While useful for contextualizing findings within the existing literature, search algorithms, inaccurate and misleading string matching, incomplete indexing, and publication biases may have influenced the results. We potentially excluded relevant studies or overemphasized certain patterns. Since we did not perform statistical testing at this stage, our approach relied purely on keyword-based hit counts. By filtering out combinations supported by fewer than three publications, we aimed to reduce spurious associations; however, this may have decreased sensitivity and led us to miss rare but specific findings reported in only a single study. These limitations underscore the importance of complementing literature-based approaches with experimental validation to ensure robustness. Finally, the observational nature of this study precludes definitive conclusions about causality. Experimental validation, such as animal models or clinical interventions, will be critical to confirm the biological significance of the identified microbial associations. Taken together, these limitations highlight the importance of cautious interpretation and pave the way for future research to address these gaps systematically.

## 5. Conclusions

In this comprehensive metagenomic analysis of stool, saliva, and interdental plaque samples from patients with obesity, we identified distinct and site-specific microbial signatures, with stronger shifts observed in oral compared to intestinal communities. These findings support the concept of an interconnected oral–gut microbiome axis contributing to systemic metabolic alterations beyond local dysbiosis.

Several taxa, including *Actinomyces* sp., *Streptococcus* spp., and *Bifidobacterium longum*, emerged as potential biomarkers that may play active roles in metabolic disease processes. Integrating oral and gut microbiome profiling could therefore improve the diagnostic precision and therapeutic stratification of obesity.

Building on known insights and considering known limitations, this study aims to systematically analyze and compare the metagenomes of stool, saliva, and plaque samples from obesity patients. By leveraging high-throughput sequencing and rigorous multi-sample integration, we seek to identify disease-specific microbial signatures and evaluate their association with obesity. Ultimately, we aspire to advance the field toward a more comprehensive and personalized understanding of obesity, paving the way for improved therapeutic strategies.

## Data Availability

The raw metagenomic sequencing data, after the removal of ambient human DNA, has been deposited in the Sequencing Read Archive (SRA) under the accession code PRJNA1057503: https://www.ncbi.nlm.nih.gov/bioproject/PRJNA1057503 (accessed on 27 December 2023).
